# Stability Performance of Inductively Coupled Plasma Mass Spectrometry-Phenotyped Kernel Minerals Concentration and Grain Yield in Maize in Different Agro-Climatic Zones

**DOI:** 10.1371/journal.pone.0139067

**Published:** 2015-09-25

**Authors:** Mallana Gowdra Mallikarjuna, Nepolean Thirunavukkarasu, Firoz Hossain, Jayant S. Bhat, Shailendra K. Jha, Abhishek Rathore, Pawan Kumar Agrawal, Arunava Pattanayak, Sokka S. Reddy, Satish Kumar Gularia, Anju Mahendru Singh, Kanchikeri Math Manjaiah, Hari Shanker Gupta

**Affiliations:** 1 Maize Research Lab, Division of Genetics, ICAR-Indian Agricultural Research Institute, New Delhi, India; 2 ICAR-Indian Agricultural Research Institute, Regional Research Centre, Dharwad, India; 3 International Crops Research Institute for the Semi-Arid Tropics, Hyderabad, India; 4 ICAR-Vivekanand Parvatiya Krishi Anusandhan Sansthan, Almora, India; 5 ICAR Research Complex for North Eastern Hill Region, Umiam, Meghalaya, India; 6 Professor Jayshankar Telangana State Agricultural University, Hyderabad, India; 7 C.S.K. Himachal Pradesh Krishi Viswavidyalaya, Bajaura, India; 8 Grain Quality Laboratory, Division of Genetics, ICAR-Indian Agricultural Research Institute, New Delhi, India; 9 Division of Soil Science and Agricultural Chemistry, ICAR-Indian Agricultural Research Institute, New Delhi, India; NBPGR, INDIA

## Abstract

Deficiency of iron and zinc causes micronutrient malnutrition or hidden hunger, which severely affects ~25% of global population. Genetic biofortification of maize has emerged as cost effective and sustainable approach in addressing malnourishment of iron and zinc deficiency. Therefore, understanding the genetic variation and stability of kernel micronutrients and grain yield of the maize inbreds is a prerequisite in breeding micronutrient-rich high yielding hybrids to alleviate micronutrient malnutrition. We report here, the genetic variability and stability of the kernel micronutrients concentration and grain yield in a set of 50 maize inbred panel selected from the national and the international centres that were raised at six different maize growing regions of India. Phenotyping of kernels using inductively coupled plasma mass spectrometry (ICP-MS) revealed considerable variability for kernel minerals concentration (iron: 18.88 to 47.65 mg kg^–1^; zinc: 5.41 to 30.85 mg kg^–1^; manganese: 3.30 to17.73 mg kg^–1^; copper: 0.53 to 5.48 mg kg^–1^) and grain yield (826.6 to 5413 kg ha^–1^). Significant positive correlation was observed between kernel iron and zinc within (r = 0.37 to r = 0.52, *p* < 0.05) and across locations (r = 0.44, *p* < 0.01). Variance components of the additive main effects and multiplicative interactions (AMMI) model showed significant genotype and genotype × environment interaction for kernel minerals concentration and grain yield. Most of the variation was contributed by genotype main effect for kernel iron (39.6%), manganese (41.34%) and copper (41.12%), and environment main effects for both kernel zinc (40.5%) and grain yield (37.0%). Genotype main effect plus genotype-by-environment interaction (GGE) biplot identified several mega environments for kernel minerals and grain yield. Comparison of stability parameters revealed AMMI stability value (ASV) as the better representative of the AMMI stability parameters. Dynamic stability parameter GGE distance (GGED) showed strong and positive correlation with both mean kernel concentrations and grain yield. Inbreds (CM-501, SKV-775, HUZM-185) identified from the present investigation will be useful in developing micronutrient-rich as well as stable maize hybrids without compromising grain yield.

## Introduction

Micronutrient malnutrition or hidden hunger is a growing concern worldwide and identified among the top priority global problems. Out of 17 micronutrients, iron (Fe) and zinc (Zn) deficiencies are the most widespread in developing countries including India [1]. Globally, one in four people are affected by Fe deficiency anaemia (IDA) especially pregnant women and preschool-age children are at highest risk. Zn deficiency is also widespread (25% of population) in the world and associated with incidence of diarrhoea, pneumonia and malaria among pre-school children [2, 3]. In addition to Fe and Zn, manganese (Mn) and copper (Cu) also important for synthesis of enzymes, hormones, vitamins, fluid regulation, cellular integrity and energy production in humans [4]. The situation is more severe in Africa and South-East Asia, where about two thirds of preschool-age children and half of all women are affected by malnutrition [5]. Widespread occurrence of malnutrition in African and South-East Asian countries is mainly due to dependency of the population largely on cereal-based diets which possess lower concentration of mineral elements [6].

Maize is a leading cereal in terms of both production (1014 million t) and productivity (4.91 t ha^–1^) contributing 34.3% of total cereal production. Sixty seven percent of its total production comes from low and lower middle income countries, signifying its vital role in the addressing the malnutrition and economy of millions of poor farmers [7]. Thus, breeding of maize cultivars with increased micronutrients concentration can fulfil the mineral-nutrition requirement of malnourished population [8]. Development of micronutrient-rich maize cultivars requires substantial and useful genetic variation for the target micronutrients. In maize, several studies show the presence of appreciable variation for kernel mineral concentrations [9–15].

Phenotype is not only manifested as a result of its own genetic composition and its surrounding environment but also their interactions [16]. Concentrations of mineral micronutrients in the kernels are also influenced by various complex factors *viz*., genotype *per se*, soil properties, and interactions among nutrients [17]. Selecting genotypes with stable expression of mineral micronutrients across testing environments is as important as increasing the concentration of these mineral micronutrients in the kernels [9]. Previous experiments reported the contribution of genotype (G) × environment (E) interaction to the expression of kernel minerals concentration [9, 13–14, 18–19], however, in most of the studies phenotyping of kernel minerals was based on less precision phenotyping platforms and no comparisons were available between hill and plain environments on Fe and Zn accumulation in maize kernels.

Several univariate (joint regression analysis (JRA) [20–22]; ecovalence measure [23]; stability variance [24]; environmental variances [25]; coefficient of variability [26]; superiority measure of genotypic performance [27]) and multivariate (AMMI [28–29]; GGE biplot [30]) statistical models are available to quantify the G × E interaction. Comparing the efficiency of different stability models will aid to select better genotypes especially for the complex traits. Hence, the assessment of stability of the maize inbred lines for kernel micronutrients status in addition to grain yield is important to select the reliable inbred lines for breeding kernel micronutrient rich maize cultivars. The present investigation aimed at evaluating stability performance of kernel micronutrients and grain yield in maize in different agro-climatic zones of India with a view to identify promising and stable inbred lines for kernel micronutrients and grain yield.

## Materials and Methods

### Genetic material

A set of 50 maize inbred lines (S1 Table) selected from various Indian breeding institutes (Acharya N. G. Ranga Agricultural University, Hyderabad; Banaras Hindu University, Varanasi; Chaudhary Charan Singh Agricultural University, Hisar; G. B. Pant University of Agricultural Science and Technology, Pantnagar; Indian Agricultural Research Institute, New Delhi; Punjab Agricultural University, Ludhiana and Vivekananda Parvatiya Krishi Anusandhan Sansthan, Almora) and CIMMYT, Mexico based on genetic diversity and place of origin (exotic or indigenous) was used in this experiment.

### Field evaluation

Trials were grown in six diverse environments of India which comprise three hill environments: Almora (29°36'N, 79°40' E; 1250 masl) Bajaura (32°20'N, 77°00'E; 1090 masl) and Barapani (25°39'N, 91° 54' E; 1010 masl) and three plain environments: Delhi (28°23'N, 77°27'E; 229 masl), Dharwad (15°26'N, 75°07'E; 678 masl) and Hyderabad (17°22'N, 78°28′E; 489 masl) during *Kharif* season, 2013 and standard agronomic practices (http://agridaksh.iasri.res.in) were followed to raise the trials. The field trials were part of the activities of the collaborative project involving all researchers of the respective locations hence no specific permission was required to carry out the trials. The soil nutrients profile of test locations given in S2 Table. All trials were conducted in randomised complete block design with three replications and phenotyped for Fe, Zn, Mn and Cu concentrations, and grain yield. Grain yield was calculated by considering fresh weight per plot, dry matter, shelling percent and moisture at 15% [31] and expressed in kg/ha.

### Sampling of kernels for micronutrients concentration

After the crop reached physiological maturity, 3 to 5 selfed cobs per entry were harvested with husk and dried under shade. Husk was removed from each ear and seeds were manually shelled by clean hands wearing contaminant-free gloves on a dust free and clean surface. Seeds were placed in a clean plastic tray and representative grain samples were sampled by quartering method. Seed samples were further dried at 40°C for 5 days in a clean, contaminant-free and un-corroded oven. Care was taken at every step to exclude dust and metal contamination. Kernels were washed quickly with 0.1 N HCl and followed by three rinse of milli-Q water to remove any possible surface contaminants and dried in hot air oven for 2 hours at 70°C. From each representative samples, 15 to 20 g of seeds were ground into fine powder using a Retsch Mill (Retsch, Mixer Mills: MM 400) with zirconium oxide grinding jar [32].

### Estimation of micronutrients concentration through inductively coupled plasma mass spectrometry (ICP-MS)

Flour samples (0.5 g) were digested in a microwave digestion system (Anton Parr: Multiwave ECO) with concentrated nitric acid (Suprapur^®^ grade, Merck, Germany) and digested samples were transferred to 50 ml volumetric flask to make up the dilution volume. The kernel micronutrients concentration were analyzed using ICP-MS platform with auto-sampling protocol (Perkin Elmer, Model: NexION 300 ICP-MS). All micronutrients concentration were computed as mg kg^–1^ kernels. Quality control and assurance was assessed using known working standard after every ten sample reading.

### Descriptive Statistics

Mean, range, correlation of kernel micronutrients and grain yield within and across locations were computed using Genstat release 16.1 [33].

### Repeatability and broad sense heritability

The repeatability h^2^ for each test location was computed as per Piepho and Mohring [34] and Broad sense heritability (H_lr_) of the entry means was computed as per Milligan et al. [35].

### Additive main effects and multiplicative interactions (AMMI) model

Magnitude of genotype, environment and genotype × environment (G × E) interaction was assessed by ANOVA using the AMMI model [36] by keeping genotype in fixed and environment in random effects. AMMI was performed by Genstat release 16.1 [33]. The AMMI model for G genotypes and E environments is as

$${\text{Y}}_{\text{ij}}=\mu +{\text{g}}_{\text{i}}+{\text{e}}_{\text{j}}+\sum_{\text{n}=1}^{\text{N}}\lambda_{\text{n}}\gamma_{\text{in}}\alpha_{\text{jn}}+\rho_{\text{ij}}+\varepsilon_{\text{ij}}$$

Where, Y_ij_ = target trait response of i^th^ genotype (i = 1, 2,…, I) in j^th^ environment j (j = 1, 2,…, J); μ = is the general mean; g_i_ = main effect associated to the i^th^ genotype; e_j_ = main effect associated to the j^th^ environment; N = max (G-1, E-1) *i*.*e* the number of principal axes (principal components) retained in the model to describe the pattern of the interaction between the i^th^ genotype with the j^th^ environment; λ_n_ = singular value of the n^th^ principal interaction axis; γ_in_ = i^th^ element of the singular column vector associated to axis n; α_jn_ is the j^th^ element of the singular row vector associated to axis n; ρ_ij_ is the AMMI residue; and ε_*ij*_ = pooled error.

### GGE biplot model

GGE biplot [37, 38] was constructed using entry means from each environment for kernel micronutrients concentration and grain yield using Genstat release 16.1 software [33]. We generated GGE biplots using following mathematical model:

$$Y_{ij}-{\bar{Y}}_{.j}=\lambda_{1}\xi_{i1}\eta_{j1}+\lambda_{2}\xi_{i2}\eta_{j2}+e_{ij}$$

Where, Y_ij_ = average yield of i^th^ genotype in j^th^ environment, ${\bar{Y}}_{.j}$ = average yield over all genotypes in j^th^ environment and λ_1_ξ_i1_η_j1_ and λ_2_ξ_i2_η_j2_ = collectively the first and second principal component (PC1 and PC2); λ_1_ and λ_2_ = singular values for the first and second principal components, PC1 and PC2, respectively; ξ_i1_ and ξ_i2_ = PC1 and PC2 scores, respectively for the i^th^ genotype; η_j1_ and η_j2 =_ PC1 and PC2 scores, respectively for j^th^ environment; and e_ij_ = residual of the model associated with the i^th^ genotype in the j^th^ environment.

### Rank correlation among different statistical models

Spearman correlation coefficient was computed among stability models for all the traits using Genstat release 16.1 [33]. Traits (grain yield and kernel micronutrients concentration) ranking as well as trait-stability ranking of Eberhart and Russell (ER), AMMI and GGE biplot (S3–S7 Tables) were used to compute the correlation coefficient [39–41].

### Comparison of stability parameters

#### Sums of the absolute value of the IPC Scores: SIPC1 and SIPCF (AMMI)

SIPC1 and SIPCF are the sum of absolute value of the IPC scores for each inbred line and calculated as,

$$\text{SIPC}=\sum_{\text{n}=1}^{\text{n}}\lambda_{\text{n}}^{0.5}\;\gamma_{\text{in}}$$

Where, N = 1 for SIPC1; for SIPCF, N was the number of IPC that were retained in the AMMI model *via F* ratio test [42]. Genotypes with low SIPC and SIPCF value is generally considered more stable across environments.

#### Averages of the squared eigenvector values: EV1 and EVF (AMMI)

EV1 and EVF were calculated as per Zobel [43]. EV1 and EVF parameters are averages of the squared eigenvector values. For EV1, N was one; for EVF, N was the number of IPCs that were retained in the AMMI model using *F* ratio test.

$$\text{EV}=\sum_{\text{n}=1}^{\text{n}}\frac{\gamma_{\text{in}}}{\text{n}}$$

#### AMMI statistic coefficient (D)

AMMI statistic coefficient (D) was calculated as per Zhang et al. [44] and is referred to as AMMI distance. $\text{D}=\sqrt{\sum_{k=1}^{N}\gamma_{is}^{2}}$ (i = 1, 2, 3, 4,…, n)

Where, D is the distance of the interaction principal component (IPC) point from the origin in space, N is the number of significant IPCs, and γ_is_ is the score of genotype i in IPC [45]. Greater the distance of the genotype from the origin of the IPCs is considered less stability. Genotype with the lowest value of the D statistic is considered the most stable [44].

#### AMMI stability value (ASV)

Purchase et al. [46] developed ASV based on the AMMI model’s interaction principal component analysis axis 1 (IPCA1) and interaction principal component analysis axis 2 (IPCA2) scores. ASV is the distance from the coordinate point to the origin in a two dimensional scattergram generated by plotting of IPCA1 score against IPCA2 score. IPCA1 score contributes more to SS_GE,_ therefore it has to be weighted by the proportional difference between IPCA1 and IPCA2 scores to compensate for the relative contribution of IPCA 1 and IPCA 2.

$${\text{ASV}}_{\text{i}}=\sqrt{{\left[\frac{{\text{SS}}_{\text{IPCA}1}}{{\text{SS}}_{\text{IPCA}2}}\right]}^{2}+{\left[\text{IPCA}2\;\text{Score}\right]}^{2}}$$

#### Genotype main effect plus genotype-by-environment interaction distance (GGED)

GGE distance generated as per Yan [47] in order to assign the rankings for each genotype. GGE distance is the distance of each genotype from the ideal genotype. Lower GGE distance score indicates the most desirable genotype.

#### Joint regression analysis (JRA) parameters

The regression coefficient (b_i_) and variance in regression deviation (S^2^_di_) was calculated as per Eberhart and Russell (ER) model [22].

## Results

### Environmental means and variance components

Descriptive statistical analysis of kernel micronutrients and grain yield revealed significant variability within and across the agro climatic zones (Table 1). Relatively, lower mean kernel micronutrients concentration (Fe: 27.18 mg kg^–1^; Zn: 9.07 mg kg^–1^; Mn: 6.66 mg kg^–1^; Cu: 1.43 mg kg^–1^) and grain yield (1920 kg ha^–1^) were recorded in Almora and Barapani, respectively as compared to other locations. Genotypes accumulated higher kernel Fe in Dharwad, kernel Zn and Mn in Delhi, kernel Cu in both Dharwad and Hyderabad and recorded higher grain yield in Hyderabad.

**Table 1:** Descriptive statistics of kernel minerals concentration and grain yield of 50 maize inbreds in six test environments.

	Fe (mg kg^–1^)				Zn (mg kg^–1^)				Mn (mg kg^–1^)				Cu (mg kg^–1^)				Grain yield (kg ha^–1^)			
Environment	Mean	S.E (±)	Min.	Max.	Mean	S.E (±)	Min.	Max.	Mean	S.E (±)	Min.	Min.	Mean	S.E (±)	Min.	Max.	Mean	S.E (±)	Min.	Max.
Almora	27.18	0.53	18.88	36.69	9.07	0.23	5.41	12.83	27.18	0.53	18.88	36.69	9.07	0.23	5.41	12.83	2506.60	133.30	1093.30	4720.00
Bajaura	32.93	0.56	24.79	45.01	17.20	0.38	11.91	23.07	32.93	0.56	24.79	45.01	17.20	0.38	11.91	23.07	2933.30	106.70	1733.30	4800.00
Barapani	32.37	0.59	25.60	41.65	17.25	0.35	13.34	24.84	32.37	0.59	25.60	41.65	17.25	0.35	13.34	24.84	1920.00	80.00	826.70	3226.60
Delhi	33.85	0.73	22.77	44.45	19.20	0.42	14.23	26.26	33.85	0.73	22.77	44.45	19.20	0.42	14.23	26.26	2480.00	106.70	880.00	5226.60
Dharwad	34.91	0.65	25.95	47.65	15.68	0.29	11.77	19.91	34.91	0.65	25.95	47.65	15.68	0.29	11.77	19.91	1653.30	53.30	960.00	2933.30
Hyderabad	31.12	0.63	24.02	44.73	18.14	0.45	11.84	30.85	31.12	0.63	24.02	44.73	18.14	0.45	11.84	30.85	3333.30	106.70	1920.00	5413.30
Grand mean	32.06	0.56	23.94	42.41	16.08	0.28	11.83	21.44	32.06	0.56	23.94	42.41	16.08	0.28	11.83	21.44	2453.30	53.30	1733.30	3733.30

All kernel micronutrients and grain yield showed moderate to high level of repeatabilities within the environment. Positive and significant correlations were observed among all environments for all kernel minerals ranging from 0.36 (Zn: between Almora and Barapani) to 0.89 (Fe: between Dharwad and Hyderabad). Significant and positive rank correlations were only found among the hill environments (Almora, Bajaura and Bararapani), between hill environments and Delhi, Dharwad and Almora, Hyderabad and Bajaura, and Hyderabad and Delhi (Table 2) for grain yield.

**Table 2:** Estimated repeatability (bold on diagonal) in each environment and phenotypic correlation among environments for kernel minerals concentration and grain yield (Fe, Mn and grain yield: below the diagonal; Zn, Cu: above the diagonal).

Env (Fe\Zn)	Almora	Bajaura	Barapani	Delhi	Dharwad	Hyderabad
Almora	**0.75/0.64**	0.56^b^	0.36^b^	0.51^b^	0.52^b^	0.58^b^
Bajaura	0.82^b^	**0.64/0.83**	0.54^b^	0.65^b^	0.62^b^	0.63^b^
Barapani	0.83^b^	0.77^b^	**0.82/0.77**	0.68^b^	0.60^b^	0.64^b^
Delhi	0.83^b^	0.79^b^	0.82^b^	**0.88/0.83**	0.75^b^	0.72^b^
Dharwad	0.88^b^	0.87^b^	0.84^b^	0.83^b^	**0.81/0.79**	0.71^b^
Hyderabad	0.82^b^	0.83^b^	0.79^b^	0.84^b^	0.89^b^	**0.82/0.88**
**Env (Mn\Cu)**						
Almora	**0.84/0.88**	0.85^b^	0.85^b^	0.74^b^	0.80^b^	0.80^b^
Bajaura	0.83^b^	**0.85/0.90**	0.87^b^	0.76^b^	0.79^b^	0.83^b^
Barapani	0.78^b^	0.83^b^	**0.75/0.57**	0.78^b^	0.84^b^	0.84^b^
Delhi	0.84^b^	0.77^b^	0.81^b^	**0.89/0.93**	0.79^b^	0.78^b^
Dharwad	0.69^b^	0.72^b^	0.69^b^	0.72^b^	**0.87/0.91**	0.84^b^
Hyderabad	0.81^b^	0.75^b^	0.75^b^	0.86^b^	0.76^b^	**0.86/0.71**
**Env (Grain yield)**						
Almora	**0.97**					
Bajaura	0.59^b^	**0.97**				
Barapani	0.64^b^	0.39^b^	**0.98**			
Delhi	0.59^b^	0.33^a^	0.39^b^	**0.98**		
Dharwad	0.31^a^	0.06	0.24	0.28	**0.94**	
Hyderabad	0.27	0.29^a^	0.11	0.39^b^	0.10	**0.97**

^a^, ^b^Significant at *p* < 0.05 and *p* < 0.01, respectively.

### Correlation among kernel micronutrients and grain yield

Correlation coefficients were computed in a pair-wise combination for all the kernel minerals and grain yield. Significant and positive correlation (*p* < 0.05) was found between kernel Fe and Zn and kernel Fe and Mn in all environments. However, coefficients of correlation between kernel Fe and Cu concentration were found non-significant. Kernel Zn concentration was also found significantly correlated with kernel Mn in all the environments except in Barapani and with kernel Cu, except in Almora and Hyderabad. Coefficients of correlation between grain yield and kernel mineral concentration showed non-significant to negative-significant values. Significant and negative correlation was observed for kernel Fe and grain yield, kernel Cu and grain yield in Dharwad and between kernel Zn and grain yield in Hyderabad (Table 3).

**Table 3:** Phenotypic correlation among kernel mineral micronutrients and grain yield across six test environments.

	Environment	Zn	Mn	Cu	Grain yield
**Fe**	Almora	0.43^c^	0.36^a^	0.14	–0.05
	Bajaura	0.37^a^	0.32^a^	0.13	–0.20
	Barapani	0.40^a^	0.39^a^	0.21	0.07
	Delhi	0.46^b^	0.40^a^	0.22	0.09
	Dharwad	0.52^c^	0.38^b^	0.18	–0.41^b^
	Hyderabad	0.40^c^	0.37^a^	0.12	–0.03
	Pooled	0.44^b^	0.35^a^	0.15	–0.06
**Zn**	Almora		0.43^c^	0.17	–0.03
	Bajaura		0.31^a^	0.33^a^	–0.05
	Barapani		0.25	0.32^a^	–0.09
	Delhi		0.37^b^	0.51^b^	–0.03
	Dharwad		0.39^b^	0.49^c^	–0.42
	Hyderabad		0.37^a^	0.15	–0.30^a^
	Pooled		0.36^b^	0.38^b^	–0.16
**Mn**	Almora			0.27	0.04
	Bajaura			0.26	0.05
	Barapani			0.37^a^	–0.09
	Delhi			0.32^a^	–0.06
	Dharwad			0.35^a^	–0.26
	Hyderabad			0.24	0.14
	Pooled			0.32^a^	0.02
**Cu**	Almora				–0.18
	Bajaura				–0.13
	Barapani				–0.10
	Delhi				–0.06
	Dharwad				–0.33^a^
	Hyderabad				0.02
	Pooled				–0.14

^a^,^b^,^c^Significant at *p* < 0.05, *p* < 0.01 and *p* < 0.001, respectively.

### Combined AMMI-ANOVA and broad sense heritability

AMMI-ANOVA was performed for kernel minerals concentration and grain yield to know the contribution of genotype, environment and G × E interaction component to the observed total variation. AMMI-ANOVA on kernel mineral concentration and grain yield showed significant contribution (*p* < 0.01) of main effects due to genotype, G × E interaction and environment components.

For kernel Fe, Mn and Cu, the major portion of the variation was contributed by genotypic effect (Fe: 39.6%; Mn: 41.3%; Cu: 41.1%) followed by G × E interaction effect (Fe: 27.5%; Mn: 23.4%; Cu: 25.8%) whereas the lowest contribution was by environmental effect (Fe: 13.8%; Mn: 19.7%; Cu: 16.8%). On contrary, the environmental effect contributed predominately to the total variation (Zn: 40.5%; grain yield: 37.0%) followed by G × E interaction effect (Zn: 26.3%; grain yield: 34.1%) and the least by genotypic effect (Zn: 21.3%; grain yield: 25.7%) for kernel Zn concentration and grain yield. The first four principle components of G × E were highly significant for all the traits. IPCA1 and IPCA2 explained the maximum portion of interaction effects ranging from 56.6% in yield to 63.3% in kernel Mn concentration. Significant contribution of IPCA1 and IPCA2 towards G × E interactions for kernel micronutrient concentrations and grain yield confirmed the adequacy of the AMMI model, and therefore, respective biplots construction was justified (Table 4). High broad-sense heritability was observed for kernel micronutrients and grain yield. Kernel Mn showed the highest broad sense heritability (89%) followed by kernel Fe and Cu (88%), kernel Zn (77%) and grain yield (72%).

**Table 4:** Pooled AMMI-ANOVA for kernel minerals concentration and grain yield at six test environments.

		Fe		Zn		Mn		Cu		Grain yield	
Source	d.f.	M.S.	%TSS	M.S.	%TSS	M.S.	%TSS	M.S.	%TSS	M.S.	%TSS
Total	899	47.50	-	27.50	-	10.46		1.34		0.12	
Rep. (Env.)	12	33.70	0.95	4.90	0.24	7.47	0.96	0.38	0.38	0.01	0.00
Treatments	299	115.70^b^	80.95	72.60^b^	87.89	26.72^b^	84.99	3.38^b^	83.82	0.35^b^	96.94
Environments	5	1178.90^b^	13.79	2002.60^b^	40.56	370.15^b^	19.69	40.57^b^	16.82	8.05^b^	37.05
Genotypes	49	345.40^b^	z39.60	107.40^b^	21.31	79.31^b^	41.34	10.12^b^	41.12	0.57^b^	25.76
Interactions	230	51.20^b^	27.56	27.90^b^	26.01	9.79^b^	23.96	1.36^b^	25.87	0.16^b^	34.13
IPCA 1	53	76.90^b^	9.53	48.30^b^	10.38	15.54^b^	8.77	1.94^b^	8.51	0.24^b^	11.77
IPCA 2	51	62.40^b^	7.44	28.10^b^	5.80	11.79^b^	6.40	1.47^b^	6.20	0.16^b^	7.55
IPCA 3	49	41.30^b^	4.74	28.30^b^	5.61	6.19^b^	3.22	1.20^b^	4.89	0.15^b^	6.63
IPCA 4	47	35.20^b^	3.87	15.50^b^	2.96	5.95^b^	2.98	1.04^b^	4.04	0.11^b^	4.93
Residuals	30	28.10^a^	1.97	10.40	1.26	8.15	2.60	0.90	2.23	0.09^b^	3.25
Error	438	17.7	18.1	6.70	11.87	3.09	14.05	0.46	15.81	0.01	2.96

^a^,^b^Significant at *p* < 0.05 and *p* < 0.01, respectively.

### Assessing the stability of genotypes based on AMMI Biplots

G × E interaction of kernel micronutrients and grain yield were analysed using AMMI biplots. AMMI 1 biplot presenting both mean grain yield and stability simultaneously were used to identify the best performing inbreds in the respective location and to make comparison with other stability statistics.

#### Kernel Fe

**Fig 1:**
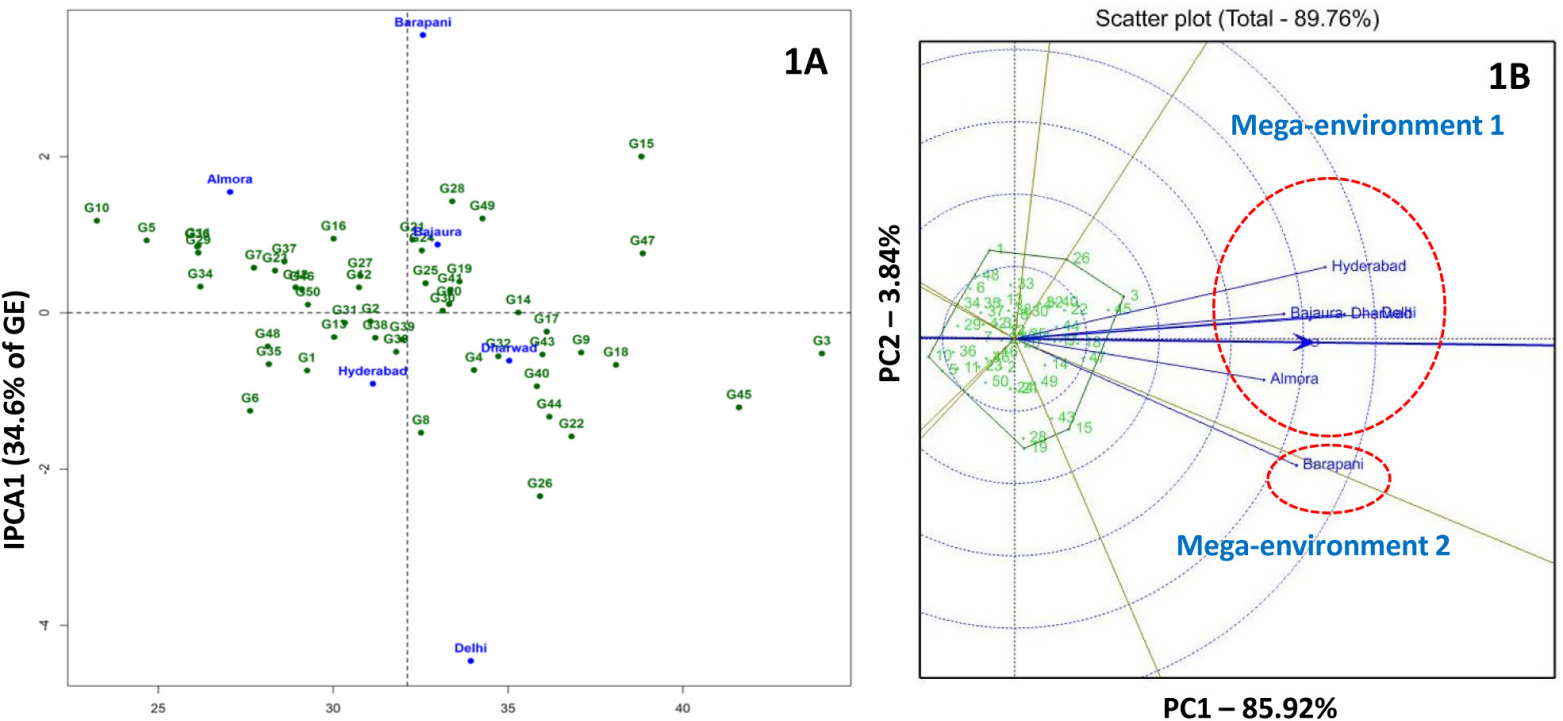
AMMI 2 (1A) and GGE (1B) biplots for kernel iron concentration in six environments. Mega-environment 1 comprises of Almora, Bajaura, Delhi and Hyderabad; Barapani falls under mega-environment 2.

For kernel Fe concentrations IPC1 accounted 34.6% of G × E interaction and 9.5% of the total variation. AMMI 1 biplots placed inbreds G14 (HKI-323) and G30 (SKV-1161) on the IPC1 zero line suggesting their suitability for general adaptation. Several inbreds with higher mean kernel concentrations found away from the IPC1 zero line was revealed that they adapted to specific environment (Fig 1A).

#### Kernel Zn

**Fig 2:**
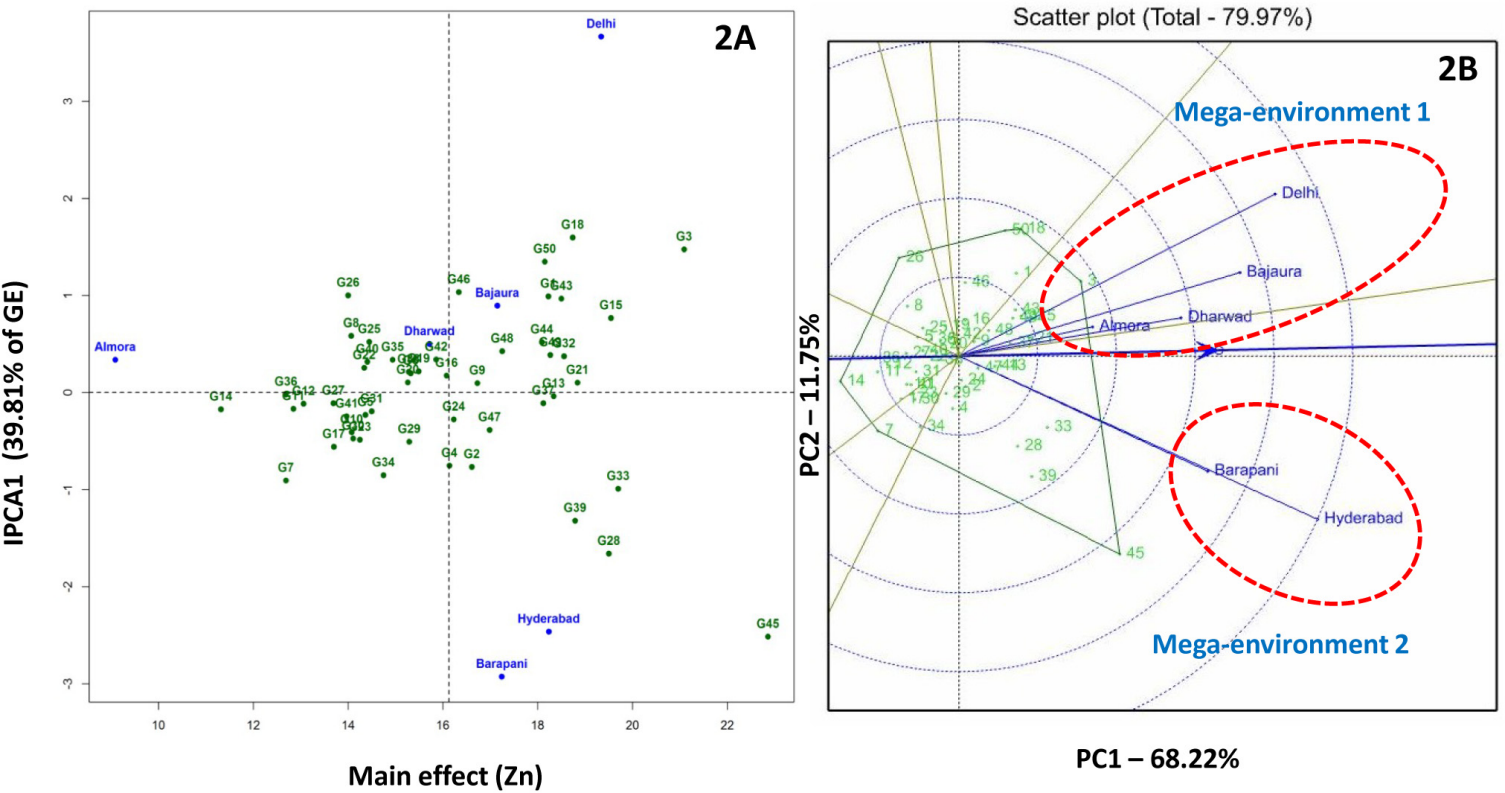
AMMI 2 (2A) and GGE (2B) biplots for kernel zinc concentration in six environments. Mega-environment 1 comprises of Almora, Bajaura and Delhi; mega-environment 2 holds Barapani and Hyderabad.

IPC1 of AMMI 1 biplot for kernel Zn concentration explained 39.9% of interaction variation and biplot as whole explained 82.2% of treatment variation. AMMI 1 biplot revealed several inbreds (G45: SKV-775, G3: CM-501) with high mean kernel Zn concentrations (> 18 mg/kg). G21 (LM-13) showed least deviation from the IPC1 zero line (0.1) and considered to be adaptable to all test environments (Fig 2A)

#### Kernel Mn

**Fig 3:**
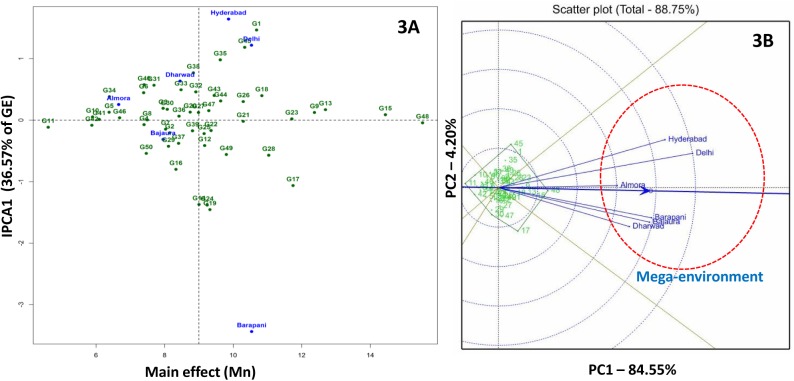
AMMI 2 (3A) and GGE (3B) biplots for kernel manganese concentration in six environments. For kernel manganese concentration all the environments grouped in a single mega-environment.

AMMI 1 biplot for kernel Mn concentration was explained 82.1% of total treatment sum of square variance with a contribution of 36.6% from IPC1 to the total G × E interaction. Based on AMMI 1 biplot, G23 (LM-16) and G48 (V-351) were found suitable for broader adaptation with high kernel Mn (Fig 3A).

#### Kernel Cu

**Fig 4:**
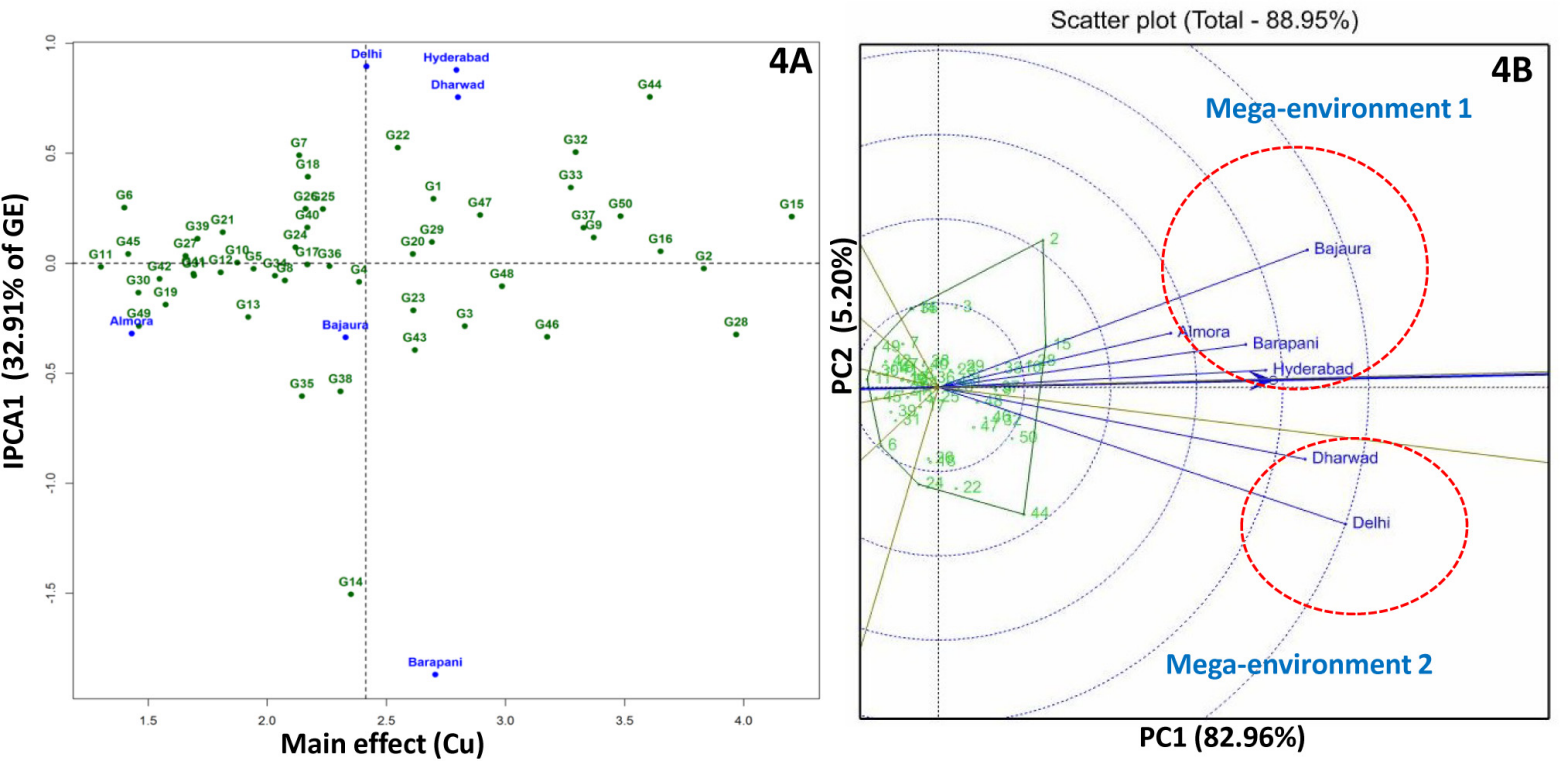
AMMI 2 (4A) and GGE (4B) biplots for kernel copper concentration in six environments. Mega-environment 1 comprises of Almora, Bajaura, Barapani and Hyderabad; Delhi and Dharwad falls under mega-environment 2.

AMMI 1 biplot of kernel Cu explained 79.8% of total treatment variation and IPC1 alone contributed 32.9% to interaction variation. G2 (CM-139) and G16 (IARI-28503) showed minimum deviation from IPC1 zero line and found stable across the locations with > 3 mg/kg kernel Cu concentration (Fig 4A).

#### Grain Yield

**Fig 5:**
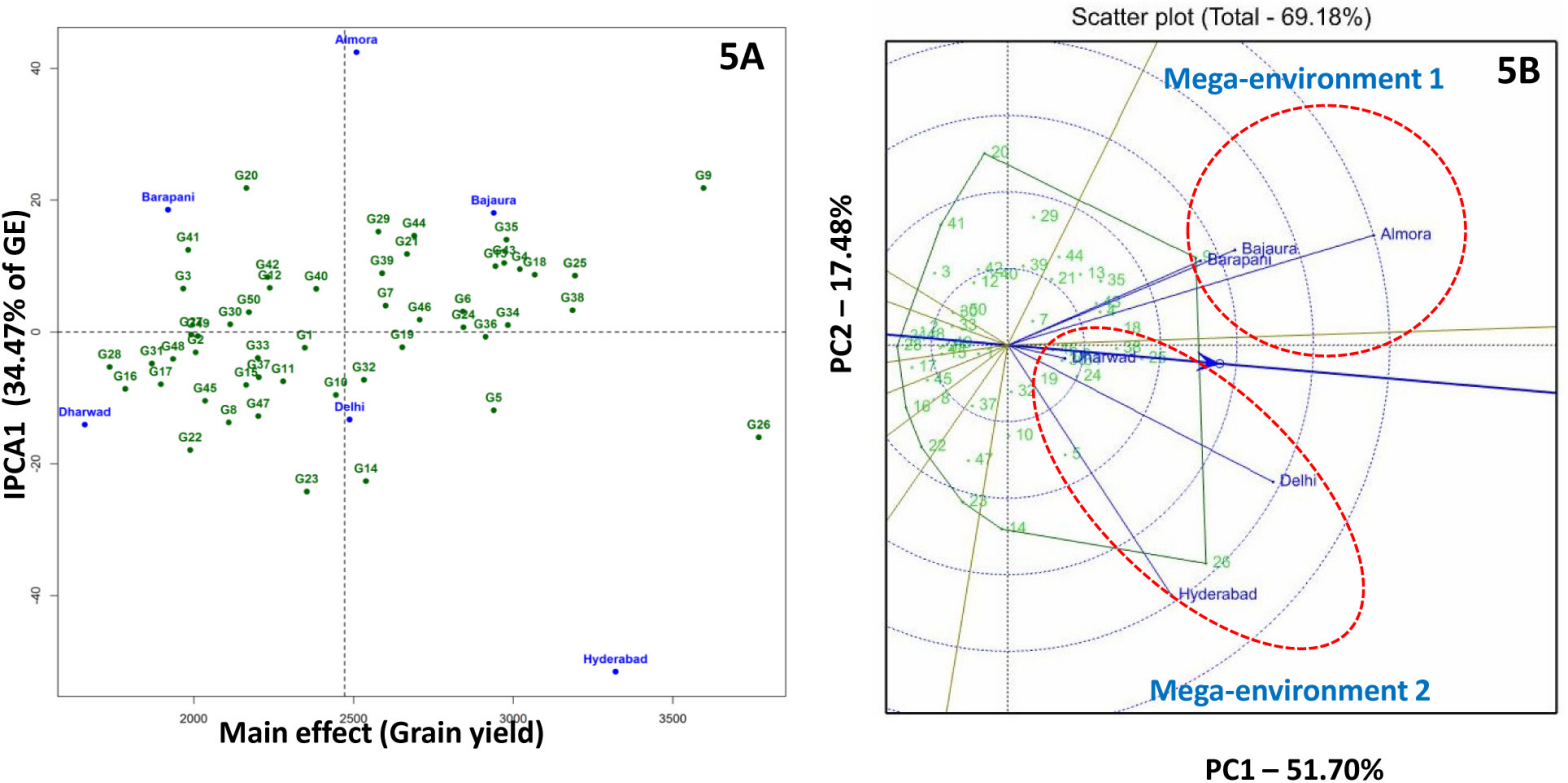
AMMI 2 (5A) and GGE (5B) biplots for grain yield in six environments. Mega-environment 1 comprises hill locations (Almora, Bajaura, Barapani); mega-environment 2 comprises plain locations (Hyderabad, Delhi and Dharwad).

AMMI 1 biplot explained 80.3% of total treatment sum of square variation with the contribution of 34.5% from IPC1 to interaction component. G6 (CML-22), G24 (Pant-100), G34 (SKV-311), G36 (SKV-512), G38 (SKV-58) and G46 (SKV-8) were identified as stable genotypes with mean grain yield of > 2.600kg ha^–1^ (Fig 5A).

### Identification of mega-environments for kernel micronutrients and grain yield

GGE biplots were generated to identify mega-environments for kernel micronutrients concentration and grain yield. For kernel micronutrient concentrations, principle component axes explained 79.9 to 89.7% of the G + G × E interaction variation (Fe: 89.7%; Zn: 79.9%; Mn: 88.7%; Cu: 88.9%). However, GGE biplot for grain yield depicted only 69.2% of the G + G × E interaction variation. GGE biplots for kernel micronutrients revealed narrow clustering of most of the test locations. This could be due to the presence of high rank correlations among environments for kernel micronutrients *i*.*e*. presence of non-crossover G × E interaction. Non-spreading of environmental vectors in GGE biplots revealed the presence of strong rank correlation between environments for kernel micronutrients concentration and non-crossover type of G × E interaction.

GGE biplot for kernel Fe concentration grouped Hyderabad, Bajaura, Delhi, Dharwad and Almora as mega-environment 1 in a single sector (sector 1) and Barapani in different sector (mega-environment 2). G3 (CM-501) and G45 (SKV-775) were found suited for mega-environment 1 and G15 (HUZM-185), G43 (SKV-671) and G49 (VQL-1) were found better fit to sector 2 (Fig 1B). Kernel Zn-GGE biplot defined two mega-environments: 1) Almora, Bajaura, Delhi and Dharwad (mega-environment 1) and 2) Barpani and Hyderabad (mega-environment 2). G3 (CM-501) and G45 (SKV-775) were the best kernel Zn accumulators in mega-environment 1 and mega-environment 2, respectively (Fig 2B).

GGE biplot for kernel Mn concentration brought all the test locations in a single sector so it could be considered as a single mega-environment. For kernel Mn concentration G48 (V-351), G15 (HUZM-185) and G13 (HKI-193-2) inbred lines were found suitable for all the locations (Fig 3B). For kernel Cu concentration two mega-environments were identified. Mega-environment 1 comprised of Almora, Bajaura, Barapani and Hyderabad and mega-environment 2 included Delhi and Dharwad. G2 (CM-139) was the best inbred for mega-environment 1 followed by G15 (HUZM-185), G28 (SE-547), G16 (IARI-28503) and G33 (SKV-247). G44 (SKV-731), G50 (VQL-2), G32 (SKV-246) and G46 (SKV-8) were the best inbreds for mega-environment 2 (Fig 4B).

GGE biplot for grain yield clearly distinguished hill and plain locations under two different sectors. G9 (HKI-1128) was the best-yielding inbred in hill mega-environment followed by G18 (IARI-28508), G4 (CML-124), G35 (SKV-38), G43 (SKV-671) and G13 (HKI-193-2). For plain mega-environment, G26 (Pant-113) was the best-yielding inbred followed by G25 (Pant-110), G38 (SKV-58), G24 (Pant-100), G5 (CML-161) and G36 (SKV-512) (Fig 5B).

### Comparison of stability models in ranking of genotypes

Three stability models were compared for their efficiency in ranking of inbred lines based on micronutrient concentration/grain yield, stability *per se* and stability-cum-micronutrient concentration/stability-cum-grain yield. Genotypes were mostly given similar rankings for kernel micronutrients concentration and grain yield by all the three stability models (S3–S7 Tables).

For stability of kernel Fe, GGE biplot ranked G7 (CML-293) as the best stable inbred and G19 (IARI-28509), the least. Both AMMI and JRA methods ranked G17 (IARI-28505), G46 (SKV-8), G41 (SKV-599), G12 (HKI-193-1), G20 (KMLD-82) and G31 (SKV-18) as the best for stability *per se*. Based on concentration-stability parameter GGE, AMMI and JRA models identified G17 (IARI-28505), G9 (HKI-1128), G41 (SKV-599), G47 (SKV-90) and G20 (KMLD-82) were the top ranked inbreds for both stability-cum-kernel Fe concentration (S3 Table). Stability ranking by all the three models identified G13 (HKI-193-2), G20 (KMLD-82), G12 (HKI-193-1) and G36 (SKV-512) as stable inbreds for kernel Zn concentration. Similarly, concentration-stability ranking by all the three stability models identified G13 (HKI-193-2), G20 (KMLD-82), G15 (HUZM-185), G9 (HKI-1128) and G21 (LM-13) as the best for both mean kernel Zn concentration-cum-stability (S4 Table). Inbred lines G5 (CML-161) and G4 (CML-124) were found as stable Mn accumulators in all stability models. For Mn concentration-cum-stability rankings, G13 (HKI-193-2) and G23 (LM-16) were found common among top ten rank across all the stability models (S5 Table).

For kernel Cu all the three models identified G20 (KMLD-82), G34 (SKV-311) and G5 (CML-161) as a top stable inbreds. Further, G16 (IARI-28503), G9 (HKI-1128), G20 (KMLD-82) and G37 (SKV-555) were identified within top ten ranks for Cu concentration-stability in all the three models (S6 Table). For grain yield stability *per se*, G27 (Pant-119) and G49 (VQL-1) were found to be the better performers in all the three stability models under investigation. Further, yield-stability ranking of inbred lines by GGE, AMMI and JRA methods identified G38 (SKV-58) as the best inbred, followed by G34 (SKV-311), G36 (SKV-512), G4 (CML-124) and G46 (SKV-8) (S7 Table).

### Rank correlation among different statistical models

Phenotypic spearman's rank correlation for kernel minerals concentration and grain yield ranks among all the three models showed almost perfect and positive (*p* < 0.01) correlation suggesting that all the three models are equally efficient in ranking of inbred lines (Table 5). However correlation between the stability ranks among the models ranges from 0.26 (NS) to 0.84 (*p* < 0.01). For kernel Fe stability and kernel Fe concentration-cum-stability ranks, significant and strong positive correlation was observed between AMMI and JRA followed by GGE and JRA, and GGE and AMMI (Table 5).

**Table 5:** Spearman’s rank correlation among three stability models in ranking of 50 maize inbreds for kernel minerals concentration/grain yield, stability and kernel minerals concentration/grain yield-stability.

Fe									
		GGE rank		AMMI rank			JRA rank		
		Stab.	Conc.-Stab.	Conc.	Stab.	Conc.-Stab.	Conc.	Stab.	Conc.-Stab.
GGE rank	Conc.	–0.06	0.67^b^	1.00^b^	–0.25	0.61^b^	0.99^b^	–0.24	0.63^b^
	Stab.		0.69^b^	–0.05	0.53^b^	0.39^b^	–0.09	0.55^b^	0.38^b^
	Conc.-Stab.			0.68^b^	0.21	0.73^b^	0.65^b^	0.23	0.74^b^
AMMI rank	Conc.				–0.23	0.62^b^	0.99^b^	–0.22	0.65^b^
	Stab.					0.61^b^	–0.30^a^	0.84^b^	0.44^b^
	Conc.-Stab.						0.55^b^	0.50^b^	0.89^b^
JRA rank	Conc.							–0.29^a^	0.60^b^
	Stab.								0.58^b^
**Zn**									
GGE Rank	Conc.	–0.36^b^	0.53^b^	1.00^b^	–0.40^b^	0.53^b^	0.97^b^	–0.40^b^	0.54^b^
	Stab.		0.57^b^	–0.35^a^	0.80^b^	0.36^b^	–0.38^b^	0.50^b^	0.08
	Conc.-Stab.			0.54^b^	0.38^b^	0.81^b^	0.50^b^	0.13	0.56^b^
AMMI Rank	Conc.				–0.38^b^	0.55^b^	0.97^b^	–0.39^b^	0.55^b^
	Stab.					0.53^b^	–0.39^b^	0.76^b^	0.28^a^
	Conc.-Stab.						0.53^b^	0.31^a^	0.73^b^
JRA Rank	Conc.							–0.37^b^	0.59^b^
	Stab.								0.51^b^
**Mn**									
GGE rank	Conc.	–0.22	0.58^b^	0.99^b^	–0.33^a^	0.58^b^	0.98^b^	–0.62^b^	0.35^a^
	Stab.		0.63^b^	–0.24	0.26	0.04	–0.22	0.53^b^	0.37^b^
	Conc.-Stab.			0.57^b^	–0.05	0.47^b^	0.58^b^	–0.03	0.59^b^
AMMI rank	Conc.				–0.35^a^	0.57^b^	0.98^b^	–0.63^b^	0.34^a^
	Stab.					0.55^b^	–0.36^a^	0.48^b^	0.17
	Conc.-Stab.						0.55^b^	–0.14	0.46^b^
JRA rank	Conc.							–0.62^b^	0.37^b^
	Stab.								0.45^b^
**Cu**									
GGE rank	Conc.	–0.15	0.65^b^	1.00^b^	–0.25	0.61^b^	0.98^b^	–0.28^a^	0.61^b^
	Stab.		0.64^b^	–0.15	0.58^b^	0.39^b^	–0.17	0.76^b^	0.47^b^
	Conc.-Stab.			0.65^b^	0.25	0.77^b^	0.62^b^	0.35^a^	0.82^b^
AMMI rank	Conc.				–0.25	0.61^b^	0.99^b^	–0.28^a^	0.61^b^
	Stab.					0.58^b^	–0.26	0.63^b^	0.26
	Conc.-Stab.						0.59^b^	0.29^a^	0.74^b^
JRA rank	Conc.							–0.27	0.61^b^
	Stab.								0.55^b^
**Grain Yield**									
GGE rank	Yield	–0.17	0.64^b^	0.98^b^	–0.11	0.67^b^	0.97^b^	–0.30^a^	0.56^b^
	Stab.		0.64^b^	–0.13	0.68^b^	0.39^b^	–0.19	0.34^a^	0.09
	Yield.-Stab.			0.65^b^	0.42^b^	0.80^b^	0.59^b^	0.03	0.50^b^
AMMI rank	Yield				–0.13	0.66^b^	0.98^b^	–0.31^a^	0.55^b^
	Stab.					0.63^b^	–0.16	0.46^b^	0.26
	Yield-Stab.						0.63^b^	0.08	0.60^b^
JRA rank	Yield							–0.30^a^	0.58^b^
	Stab.								0.57^b^

^a^, ^b^Significant at *p* < 0.05 and *p* < 0.01, respectively.

For kernel Zn concentration, GGE and AMMI models were found strongly correlated in ranking the genotypes based on stability as well as kernel Zn concentration-stability ranks followed by AMMI and JRA model. However, between GGE and JRA the strength of correlation was moderate (r = 0.50, *p* < 0.01) (Table 5). No significant correlation was observed between AMMI and GGE biplots for stability rankings of kernel Mn concentration. However, a moderate positive and significant correlation was observed between GGE and JRA (r = 0.53, *p* < 0.01) and AMMI and JRA (r = 0.48, *p* < 0.01) (Table 5).

For kernel Cu concentration, strong positive correlation was observed between GGE and JRA models for stability as well as concentration-stability ranks (r = 0.76, *p* < 0.01; r = 0.82, *p* < 0.01) followed AMMI and JRA (r = 0.63, *p* < 0.01; r = 0.74, *p* < 0.01), and GGE and AMMI (r = 0.58, *p* < 0.01; r = 0.77, *p* < 0.01) (Table 5). Further, for grain yield, GGE and AMMI models strongly correlated in assigning the ranks for both stability and grain yield-stability ranks (r = 0.68, *p* < 0.01; r = 0.80, *p* < 0.01) as compared to GGE and JRA (r = 0.34, *p* < 0.01; r = 0.50, *p* < 0.01), and AMMI and JRA (r = 0.46, *p* < 0.01; r = 0.60, *p* < 0.01) (Table 5). To sum-up, for kernel Zn concentration and grain yield, GGE ranks better reflected the AMMI results than JRA model, whereas, for kernel Fe AMMI model represented JRA results better than GGE. For kernel Mn and Cu, GGE ranks better depicted the JRA results than AMMI.

### Relationship among mean kernel micronutrients concentration and stability parameters

Genotype ranks based on *per se* mean of target traits and stability parameters were used to compute the Spearman’s rank correlation coefficient. Here, we selected mean concentration of minerals/grain yield, six AMMI parameters [sums of the absolute value of the IPC scores SIPC1 and SIPCF, averages of the squared eigenvector values EV1 and EVF, AMMI statistic coefficient (D) and AMMI’s stability value (ASV)], GGE distance from GGE biplot, regression coefficient b and variance deviation (S^2^_d_) of JRA. Inbreds were ranked based on mean concentration of kernel mineral/grain yield and stability parameters [41] (S8–S12 Tables). Significant and positive correlation was observed among all the AMMI stability parameters for all the kernel micronutrients and grain yield.

Further, within AMMI parameters correlation between SIPC1 and EV1, SIPCF and EVF, SIPCF and D, and EVF and D were found stronger (r > 0.9, *p* < 0.01). Similarly, significant and positive correlation was observed between mean kernel minerals concentration/yield and GGED for all the traits (Table 6). For kernel Fe concentration, no significant correlation was observed between mean Fe concentration and AMMI parameters, and GGED and AMMI parameters. It explains the possibility of selecting inbred with relative stable performance and high kernel Fe concentration. However, all the AMMI parameters significantly correlated with *S* ^*2*^_*d*_ parameter of JRA. Regression coefficient b was found significantly correlated with SIPC1, SIPCF and EV1 (*p* < 0.05) (Table 6). Mean Zn concentration was negatively correlated (*p* < 0.05) with SIPC1, EV1, EVF and ASV but no association was found with other stability parameters of AMMI. Moderate negative correlation was observed between kernel Zn concentration and S^2^_d_ of JRA suggesting that stable performance associated with moderately lower level of kernel Zn concentration (Table 6).

**Table 6:** Spearman's rank correlation coefficient among kernel minerals concentration/grain yield and stability parameters (^a^, ^b^Significant at *p* < 0.05 and *p* < 0.01, respectively).

Fe	SIPC1	SIPCF	EV1	EVF	D	ASV	GGED	b	S^2^_d_
Conc.	–0.11	–0.23	–0.13	–0.24	–0.23	–0.23	0.68^b^	0.06	–0.22
SIPC1		0.63^b^	0.97^b^	0.52^b^	0.64^b^	0.81^b^	0.13	0.28^a^	0.61^b^
SIPCF			0.59^b^	0.96^b^	0.98^b^	0.80^b^	0.19	0.29^a^	0.91^b^
EV1				0.48^b^	0.61^b^	0.78^b^	0.12	0.31^a^	0.58^b^
EVF					0.96^b^	0.71^b^	0.17	0.24	0.88^b^
D						0.83^b^	0.20	0.28	0.93^b^
ASV							0.21	0.20	0.84^b^
GGED								0.00	0.23
b									0.01
**Zn**									
Conc.	–0.38^b^	–0.21	–0.42^b^	–0.29^a^	–0.26	–0.38^b^	0.54^b^	0.12	–0.39^b^
SIPC1		0.69^b^	0.94^b^	0.62^b^	0.68^b^	0.89^b^	0.41^b^	0.05	0.67^b^
SIPCF			0.60^b^	0.91^b^	0.96^b^	0.81^b^	0.34^a^	0.37^b^	0.86^b^
EV1				0.53^b^	0.60^b^	0.86^b^	0.38^b^	–0.01	0.60^b^
EVF					0.93^b^	0.67^b^	0.14	0.28	0.85^b^
D						0.80^b^	0.26	0.33^a^	0.91^b^
ASV							0.38^b^	0.17	0.76^b^
GGED								0.19	0.13
b									0.14
**Mn**									
Conc.	–0.16	–0.46^b^	–0.16	–0.45^b^	–0.44^b^	–0.35^a^	0.57^b^	0.08	–0.63^b^
SIPC1		0.47^b^	0.93^b^	0.48^b^	0.49^b^	0.69^b^	0.04	0.29^a^	0.16
SIPCF			0.46^b^	0.93^b^	0.97^b^	0.79^b^	–0.18	0.32^a^	0.73^b^
EV1				0.46^b^	0.50^b^	0.67^b^	0.03	0.38^b^	0.10
EVF					0.93^b^	0.77^b^	–0.20	0.38^b^	0.68^b^
D						0.83^b^	–0.16	0.36^a^	0.71^b^
ASV							–0.05	0.37^b^	0.48^b^
GGED								0.13	–0.03
b									0.01
**Cu**									
Conc.	–0.30^a^	–0.25	–0.30^a^	–0.23	–0.27	–0.25	0.65^b^	–0.20	–0.28^a^
SIPC1		0.73^b^	0.92^b^	0.61^b^	0.69^b^	0.93^b^	0.12	0.38^b^	0.57^b^
SIPCF			0.74^b^	0.94^b^	0.98^b^	0.79^b^	0.39^b^	0.49^b^	0.81^b^
EV1				0.65^b^	0.74^b^	0.89^b^	0.16	0.39^b^	0.65^b^
EVF					0.96^b^	0.68^b^	0.40^b^	0.47^b^	0.80^b^
D						0.76^b^	0.38^b^	0.48^b^	0.83^b^
ASV							0.25	0.43^b^	0.63^b^
GGED								0.19	0.35^a^
b									0.25
**Grain Yield**									
Grain Yield	–0.14	–0.17	–0.15	–0.08	–0.16	–0.13	0.65^b^	0.16	–0.31^a^
SIPC1		0.53^b^	0.97^b^	0.46^b^	0.52^b^	0.93^b^	0.50^b^	0.14	0.40^b^
SIPCF			0.48^b^	0.90^b^	0.95^b^	0.64^b^	0.17	0.23	0.69^b^
EV1				0.41^b^	0.49^b^	0.90^b^	0.48^b^	0.19	0.36^b^
EVF					0.94^b^	0.59^b^	0.18	0.19	0.69^b^
D						0.63^b^	0.18	0.28	0.70^b^
ASV							0.42^b^	0.20	0.46^b^
GGED								0.23	0.03
b									–0.23

AMMI parameters SIPCF, EVF, D and ASV were negatively correlated with mean kernel Mn concentration and positively with S^2^_d_ of JRA. However, no significant correlation was found between AMMI parameters and GGED for kernel Mn concentration (Table 6). On the other hand, kernel Cu concentration was positive and significant among AMMI parameters and also between AMMI parameters with both b and S^2^_d_ although there was no correlation between b and S^2^_d_ (Table 6). No correlation was observed between grain yield and AMMI parameters as well as between AMMI parameters and b (Table 6)_._

## Discussion

### Exotic and Indian maize inbreds revealed significant genetic variability for kernel minerals concentration

Extensive phenotyping of 50 diverse inbred lines selected from various international and national institutes based on multi-location trials showed the presence of ample variability for kernel minerals concentration and grain yield. The extent of variation suggested that the genes responsible for kernel micronutrients accumulation were available within the maize germplasm and therefore could be used for improving kernel minerals concentration through appropriate breeding strategies.

Variability studies across six diverse environments identified inbreds with high and low minerals concentration. For kernel Fe and Zn, CM-501 and SKV-775 recorded highest concentration, whereas inbred lines HKI-161 recorded the lowest concentration. Similarly for both kernel Mn and Cu, HKI-163 and HUZM-185 can be used to derive the transgressive segregants and develop segregating mapping population. Several studies reported the polygenic inheritance of kernel micronutrient concentration [48–50]. Selective inter-mating followed by selection, recurrent selections and marker-assisted selection of target QTLs could be employed to increase the kernel micronutrients concentration. Present investigation revealed the presence of higher kernel Fe in the selected genotypes as compared to kernel Zn. These results are in accordance with that of Banziger and Long [10] and Simic et al. [51]. On the contrary high kernel Zn concentration was reported by Chakraborti et al. [13].

### Kernel Fe and Zn could be improved simultaneously

Significant phenotypic correlation among kernel micronutrients suggested the possibility for simultaneous genetic improvement of kernel micronutrient traits in maize through appropriate breeding strategies. Positive and significant correlation exist between kernel Fe and kernel Zn (r = 0.37 to 0.52) and between kernel Fe and kernel Mn (r = 0.32 to 0.40) from the multi-location trials. However, the strength of correlation varies among the environment. These results suggested that correlation among the minerals is under the control of both genetic and environmental factors. The genetic basis of the correlation among kernel minerals could be due to co-segregation of mineral transporter genes in inbred lines and/or presence of common transporters for multiple minerals [52]. Several studies have also reported the presence of positive correlation among minerals including Fe and Zn [9, 12, 53].

### Stability models revealed significant contribution of genotypic and interaction main effects

Stable performance of genotype for the target trait(s) is the key requirement in germplasm enhancement and wider adaptation for cultivation. Present investigation revealed the presence of significant G × E interaction for all the traits in six target environments which includes both hill and plain locations and lead to identification of mega-environment(s) for each trait under study. Significant level of G × E interaction for kernel minerals and grain yield in maize were also reported by Gregorio [18], Oikeh et al. [9] and Prasanna et al. [19].

The AMMI-ANOVA revealed large proportion of variation due to genotype component for kernel Fe, Mn and Cu. However, environmental contribution to the total variation was lesser than genotypic and interaction effect for kernel Fe, Mn and Cu concentration except for kernel Zn and grain yield. These results explained that kernel Zn and grain yield are much sensitive to the environmental factors as compared to kernel Fe, Mn and Cu. Significant contribution to the source of variation from genotype for grain Fe concentration and environment for grain yield and kernel Zn were also reported by Bashir et al. [54] and Menkir [12].

High heritability coupled with positive and significant correlation among all environments suggested that G × E interaction for kernel minerals is mainly a non-crossover type and it is further depicted as close grouping pattern of environments in GGE biplots. On the contrary, non-significant to significant correlation among the test environments for grain yield resulted in crossover type G × E interaction with widespread environmental vectors on GGE biplot. Minerals are the basic requirement for most of the metabolic activities including photosynthesis. Hence, plants were evolved to maintain optimal level of all mineral nutrients as the first criteria in order to ensure basic survival over other agronomically important traits such as grain yield. Therefore, this could be the underlying reason for non-crossover interaction and better heritability for kernel micronutrients as compared to crossover interaction and lower heritability for grain yield. Significant proportions of G and G × E components to the total variation further suggested that selection approaches can be employed to breed inbreds with both high kernel minerals concentration and grain yield simultaneously.

### Mega-environments were identified for kernel minerals and grain yield

A maximum of two mega environments were identified for all the traits except for kernel Mn concentration where all the environments under study were considered as single mega-environment. For grain yield, hill and plain environments were grouped into separate mega-environments. Contrastingly, for kernel micronutrients there was no clear-cut grouping of hill and plain environments and thus, suggested that stability of grain yield is more influenced by soil, altitude and other environmental factors as compared to kernel minerals. This also depicted in terms of closeness of respective environmental vectors in GGE biplot.

Inbred lines G9 (HKI-1128) and G18 (IARI-28508) were found as best grain yielder in mega-environment 1 (Almora, Bajaura and Barapani). Genotypes G26 (Pant-113) and G25 (Pant-110) were found best suited for plain locations (Hyderabad, Delhi and Dharwad). G3 (CM-501) was the best kernel Fe and Zn accumulator both in plain (Delhi, and Dharwad) and hill (Bajaura and Almora). Similarly G15 (HUZM-185) was found to accumulate higher kernel Fe, Mn and Cu in Barapani. These best fit genotypes in respective environments for the target traits could be used by the researchers for development of location-specific hybrids [55].

### Minerals concentration could be improved without compromising grain yield

Both grain yield and kernel minerals concentration are complex traits affected by genetic and non-genetic factors, including genotype, soil properties, environmental conditions and interactions of genes [56]. Correlation between grain yield and kernel minerals concentration in most of the locations under investigation were found non-significant. Banzigar and Long [10] also reported non-significant correlation between kernel Fe/Zn concentration and grain yield in maize landraces, cultivars and germplasm pools. Hence, there could be the possibility to improve the kernel micronutrients and grain yield simultaneously. Significant negative correlation was found between kernel Fe and grain yield, kernel Cu and grain yield in Dharwad environment, and between kernel Zn and grain yield in Hyderabad. However, the observation was location-specific and was noticed only in two environments out of six locations. Hence, kernel minerals including Fe and Zn in maize could be genetically improved without compromising grain yield in many of the testing locations.

Inbreds G9 (HKI-1128) and G13 (HKI-193-2) possess moderately high amount of each kernel minerals and grain yield with relative stable performance across the locations. These inbreds could therefore, be used in breeding programme for simultaneous improvement of kernel minerals and grain yield. Since, the traits are polygenically controlled, selective inter-mating followed by selection or recurrent selection are the better approaches to capture all the superior alleles for kernel micronutrients and grain yield.

### AMMI and GGE biplot models found superior to JRA model

JRA [20], GGE biplot [30, 47] and other models have been proposed to quantify the G × E interaction patterns and to identify the stable and high yielding genotypes in plant breeding programs. However, JRA, AMMI and GGE biplot are widely used in today’s context. Here, the comparison of stability models was performed employing rank correlation from JRA, GGE and AMMI models. Positive and significant correlations were observed among all stability models for mean yield or kernel minerals concentration, stability and mean yield or kernel minerals concentration-cum-yield (Table 5). However, the strength of correlation varies between the stability models. For kernel Mn and Cu concentrations, GGE and JRA models were found highly correlated as compared to GGE and AMMI or AMMI and JRA. Goyal et al. [57] also reported JRA and GGE were highly correlated while identifying stable triticale genotypes for high yield. On other hand, AMMI and JRA were found highly correlated for kernel Fe concentration as compared to GGE and AMMI for kernel Zn concentration and grain yield.

Strong and positive rank correlation for stability and mean kernel minerals concentration/grain yield-stability were observed between GGE and AMMI models in ranking the stable genotypes for kernel Zn concentration and grain yield. These suggested that both GGE and AMMI models are equally effective when contribution of environment variation is higher than the total variation. Advantage of AMMI and GGE models over JRA models were also reported in other cereal crops including wheat [41, 58] and maize [59]. Out of three models, GGE biplot and AMMI showed similar results in ranking genotypes for grain yield [60]. Although all the three methods show almost similar results with high correlation coefficient, AMMI and GGE biplot models explained better understanding of G × E interaction over JRA in our experiment.

### Stability parameters grouped into static and dynamic parameters

Correlation coefficient was computed to derive association among stability parameters, grain yield/kernel minerals concentration. The present investigation revealed association among ten stability parameters and grouped them as static and dynamic stability parameters [61] (S1 Fig). Static stability is based on environmental variance of the genotypes which is detected as all deviations from the genotypic mean. Concept of static stability is useful when the stability of line *per se* is more important; however, it may be associated with relatively poor yielding ability of genotypes although it possesses stable expression.

In the static stability parameters of AMMI model, strong and positive correlation was found among AMMI stability parameters especially among SIPCF, EVF and D; and SIPC1 and EV1 suggested that they were almost comparable in discriminating the genotypes according to their stability levels for kernel minerals concentration and grain yield across the test environments. Hence, any of these AMMI parameters could be used as an alternative for assessing the stability of the genotypes [54]. ASV showed positive and significant correlation with all other AMMI parameters. Therefore, among AMMI parameters ASV is the better representative stability parameter for AMMI analysis. Positive and significant correlation between ASV and other AMMI parameters have also been reported in other cereal crops [41, 54].

Dynamic stability parameters detect the stability of genotypes for yield and other quantitatively inherited traits [61] assuming genotype's performance will be consistent to the changes in the environment. In the present investigation, GGED and grain yield/kernel micronutrients follow dynamic stability parameters. Strong and positive correlation was observed between GGED and mean kernel minerals concentration and grain yield and were grouped together in PCoA analysis (S1 Fig). Presence of both G and G × E in GGED probably increased the heritable portion of the phenotype which was nearer to the mean performance of genotype for any target traits. The association of mean grain yield with GGED was also supported by previous reports [41, 54].

## Conclusion

The present investigation reports the presence of significant genetic variability for minerals concentration and grain yield from multi-location trials. The study also identified stable inbreds and mega environments for those traits to realise the genetic potential. The identified inbreds for high kernel Fe and Zn in the present investigation could serve as potential genetic resource for improvement of kernel Fe and Zn in maize without compromising grain yield. The presence of significant portion of heritable variation also suggested that stable maize hybrids can be developed through appropriate selection strategies for the micronutrients.

## Supporting Information

S1 Fig Principle co-ordinate analysis of stability parameters used to assess the stability of inbred lines for kernel minerals and grain yield. (TIF)

S1 Table Genotypes used for studying stability of kernel micronutrients and grain yield. (XLSX)

S2 Table Descriptions of the environment and soil nutrient profiles of the test environments. (XLSX)

S3 Table Kernel Fe concentration ranks, stability ranks and concentration-stability ranks given by each statistical method (GGE biplot, AMMI and joint regression) for 50 genotypes across six tested environments. (XLSX)

S4 Table Kernel Zn concentration ranks, stability ranks and concentration-stability ranks given by each statistical method (GGE biplot, AMMI and joint regression) for 50 genotypes across six tested environments. (XLSX)

S5 Table Kernel Mn concentration ranks, stability ranks and concentration-stability ranks given by each statistical method (GGE biplot, AMMI and joint regression) for 50 genotypes across six tested environments. (XLSX)

S6 Table Kernel Cu concentration ranks, stability ranks and concentration-stability ranks given by each statistical method (GGE biplot, AMMI and joint regression) for 50 genotypes across six tested environments. (XLSX)

S7 Table Grain yield ranks, stability ranks and yield-stability ranks given by each statistical method (GGE biplot, AMMI and joint regression) for 50 genotypes across six tested environments. (XLSX)

S8 Table Mean kernel Fe concentration (mg kg^–1^) and estimates of stability parameters along with respective ranks for 50 genotypes tested in six tested environments. (XLSX)

S9 Table Mean kernel Zn concentration (mg kg^–1^) and estimates of stability parameters along with respective ranks for 50 genotypes tested in six tested environments. (XLSX)

S10 Table Mean kernel Mn concentration (mg kg^–1^) and estimates of stability parameters along with respective ranks for 50 genotypes tested in six tested environments. (XLSX)

S11 Table Mean kernel Cu concentration (mg kg^–1^) and estimates of stability parameters along with respective ranks for 50 genotypes tested in six tested environments. (XLSX)

S12 Table Mean grain yield (kg ha^–1^) and estimates of stability parameters along with respective ranks for 50 genotypes tested in 6 tested environments. (XLSX)
