# Correction: Stability Performance of Inductively Coupled Plasma Mass Spectrometry-Phenotyped Kernel Minerals Concentration and Grain Yield in Maize in Different Agro-Climatic Zones

**DOI:** 10.1371/journal.pone.0140947

**Published:** 2015-10-15

**Authors:** Mallana Gowdra Mallikarjuna, Nepolean Thirunavukkarasu, Firoz Hossain, Jayant S. Bhat, Shailendra K. Jha, Abhishek Rathore, Pawan Kumar Agrawal, Arunava Pattanayak, Sokka S. Reddy, Satish Kumar Gularia, Anju Mahendru Singh, Kanchikeri Math Manjaiah, Hari Shanker Gupta

In Table 1, the data provided in the Fe and Zn columns are inadvertently repeated under the Mn and Cu columns. Please see the corrected Table 1 here.

**Table 1:** Descriptive statistics of kernel minerals concentration and grain yield of 50 maize inbreds in six test environments.

	Fe (mg kg^–1^)				Zn (mg kg^–1^)				Mn (mg kg^–1^)				Cu (mg kg^–1^)				Grain yield (kg ha^–1^)			
Environment	Mean	S.E (±)	Min.	Max.	Mean	S.E (±)	Min.	Max.	Mean	S.E (±)	Min.	Min.	Mean	S.E (±)	Min.	Max.	Mean	S.E (±)	Min.	Max.
Almora	27.18	0.53	18.88	36.69	9.07	0.23	5.41	12.83	6.66	0.24	3.30	10.73	1.43	0.08	0.53	2.81	2506.60	133.30	1093.30	4720.00
Bajaura	32.93	0.56	24.79	45.01	17.20	0.38	11.91	23.07	8.01	0.29	3.99	15.85	2.34	0.12	1.32	5.48	2933.30	106.70	1733.30	4800.00
Barapani	32.37	0.59	25.60	41.65	17.25	0.35	13.34	24.84	10.39	0.29	6.42	16.80	2.68	0.10	1.61	4.30	1920.00	80.00	826.70	3226.60
Delhi	33.85	0.73	22.77	44.45	19.20	0.42	14.23	26.26	10.51	0.35	5.67	17.47	2.41	0.13	0.92	4.78	2480.00	106.70	880.00	5226.60
Dharwad	34.91	0.65	25.95	47.65	15.68	0.29	11.77	19.91	8.40	0.26	4.46	12.89	2.78	0.12	1.41	4.50	1653.30	53.30	960.00	2933.30
Hyderabad	31.12	0.63	24.02	44.73	18.14	0.45	11.84	30.85	9.87	0.31	5.40	17.73	2.78	0.10	1.70	4.75	3333.30	106.70	1920.00	5413.30
Grand mean	32.06	0.56	23.94	42.41	16.08	0.28	11.83	21.44	8.97	0.26	4.87	14.93	2.41	0.09	1.38	3.92	2453.30	53.30	1733.30	3733.30
