# Supplementary figures and images for: Stability Performance of Inductively Coupled Plasma Mass Spectrometry-Phenotyped Kernel Minerals Concentration and Grain Yield in Maize in Different Agro-Climatic Zones
Source: PLoS One. 2015 Sep 25;10(9):e0139067. doi: 10.1371/journal.pone.0139067 (PMC4583500; doi:10.1371/journal.pone.0139067)

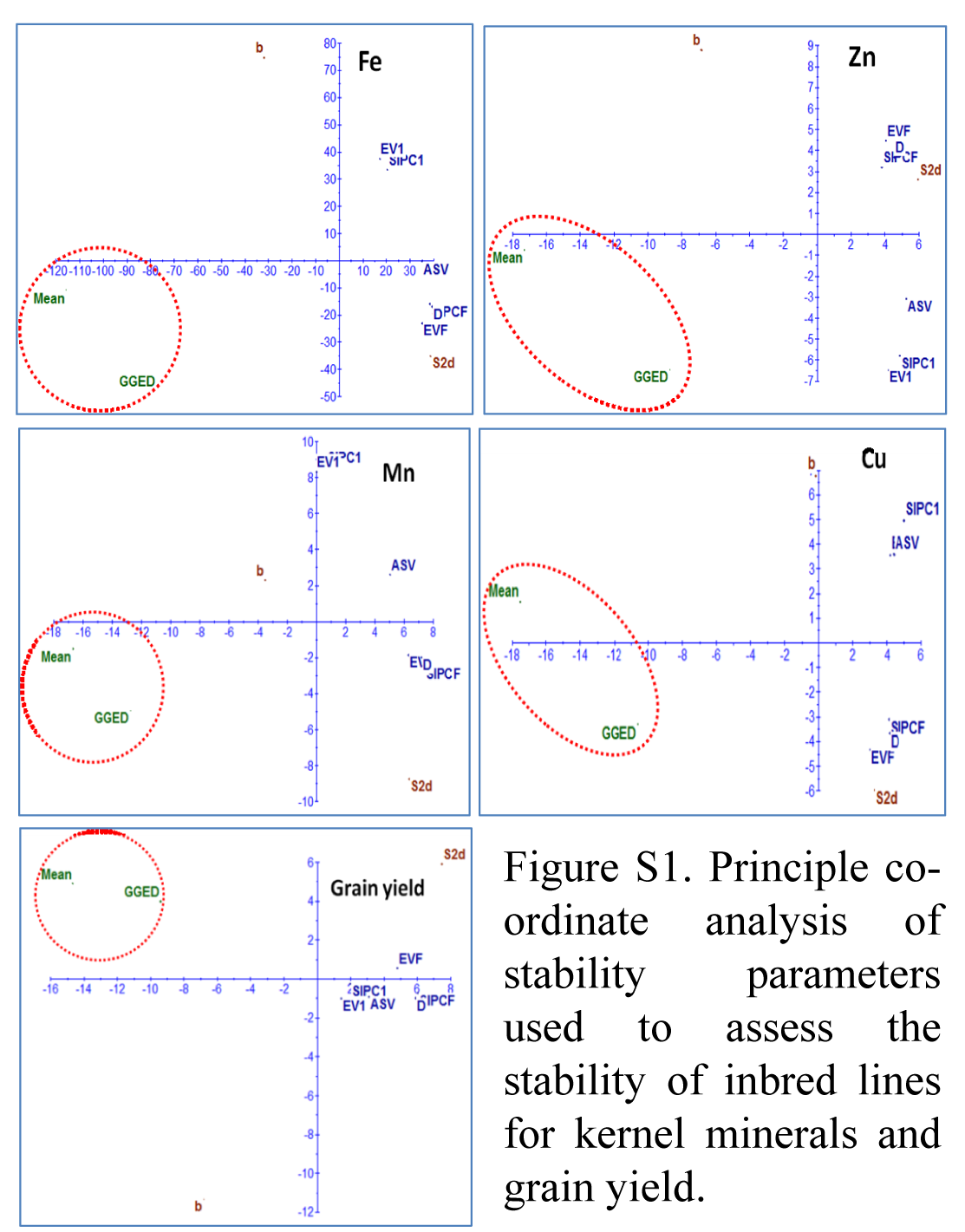

Supplement: S1 Fig — (TIF) [file pone.0139067.s001.tif]
